# Progress of the Proposed State Faculty Bill

**Published:** 1883-11

**Authors:** 


### Progress of the Proposed State Faculty Bill.

Our readers will be pleased to learn that the plan proposed by our county society for the establishment of a State Medical Faculty is receiving the hearty endorsement of the profession. Although our local society has taken the initiative in the case, it has only been to invite the co-operation of the entire profession, and there are already signs that this greatly-to-be desired co-operation is forthcoming. Copies of the proposed bill have been sent to the proper officers of each county medical society throughout the State, and in a few instances regular meetings have already been held, the matter duly presented, resolutions of endorsement adopted, and committees of co-operation appointed. Added to this, the general tone of criticism of the measure is most encouraging.

Under the head of correspondence we publish some communications received upon this subject; and we gladly avail ourselves of the opportunity to give editorial prominence to the tersely expressed opinions of an eminent member of the profession from the central portion of the State. We deem these earnest thoughts worthy of all prominence for the reason that they concisely and happily state the entire case. The correspondent writes:

"I have read the act to establish a State Faculty in Medicine, very carefully, and fully endorse it. From the opening of the code controversy, I have all the more heartily joined in the effort to conform our code to our State laws in respect to consultations, because I hoped and believed that it would drive our profession unanimously to the position of demanding of the legislature the establishment by law of one uniform, authoritative test of the proper qualifications to practice medicine, viz.: the ascertainment, by a competent board of examiners, of the possession of the requisite knowledge to be fit therefor. The questions of opinions, theories, judgments, skill, are personal, ex necessitate. But if there is any such thing as knowledge in our profession, this should be universal, and a lack of it should prevent a license to practice. So far as I have been able to discover the opinions of the members of our county society upon this proposed bill, we are unanimously in its favor. The proposition is the true and only harmonizer, and the most decisive mark of advancement. Its effect will be to clear up more rapidly the existing status, and to exercise a wholesome check upon the swarming annually of such an unnecessary number as now from our multitudinous colleges."

This is but a type of many letters which the committee have received. The writer, it will be seen, is an advocate of the new code; but the most hopeful feature in the case, and one which is an augury of success, is that the learned and most distinguished members of the profession, irrespective of any party affiliation, give this movement their hearty support. In witness of this, we call attention to the letter from the Nestor of the profession in Western New York, Prof. E. M. Moore. No man in the State or in the country has given the subject of medical education more thought than has this sagacious and eminent teacher, and we bespeak for his communication a careful perusal. Such letters, from such men, prove the truth of our statement while writing upon the subject in the last number of the JOURNAL, when we said that "our whole profession is seriously in earnest in this business, and willing, in many instances, to drop individual preferences rather than risk the chance of co-operating on the main question."

